# Predictors of Extracorporeal Membrane Oxygenation Support for Children with Acute Myocarditis

**DOI:** 10.1155/2017/2510695

**Published:** 2017-05-11

**Authors:** Han-Ping Wu, Mao-Jen Lin, Wen-Chieh Yang, Kang-Hsi Wu, Chun-Yu Chen

**Affiliations:** ^1^Division of Pediatric General Medicine, Department of Pediatrics, Chang Gung Memorial Hospital at Linko, Kweishan, Taoyuan, Taiwan; ^2^College of Medicine, Chang Gung University, Taoyuan, Taiwan; ^3^Division of Cardiology, Department of Medicine, Taichung Tzu Chi Hospital, The Buddhist Tzu Chi Medical Foundation, Taichung, Taiwan; ^4^Department of Medicine, School of Medicine, Tzu Chi University, Hualien, Taiwan; ^5^Department of Pediatric Emergency Medicine, Changhua Christian Children's Hospital, Changhua, Taiwan; ^6^School of Medicine, Kaohsiung Medical University, Kaohsiung, Taiwan; ^7^School of Post-Baccalaureate Chinese Medicine, College of Chinese Medicine, China Medical University, Taichung, Taiwan; ^8^Department of Hemato-Oncology, Children's Hospital, China Medical University Hospital, China Medical University, Taichung, Taiwan

## Abstract

The clinical presentation of acute myocarditis in children may range from asymptomatic to sudden cardiac arrest. This study analyzed the clinical spectrum of acute myocarditis in children to identify factors that could aid primary care physicians to predict the need for extracorporeal membrane oxygenation (ECMO) earlier and consult the pediatric cardiologist promptly. Between October 2011 and September 2016, we retrospectively analyzed 60 patients aged 18 years or younger who were admitted to our pediatric emergency department with a definite diagnosis of acute myocarditis. Data on demographics, presentation, laboratory tests, electrocardiogram and echocardiography findings, treatment modalities, complications, and long-term outcomes were obtained. During the study period, 60 patients (32 male, 28 female; mean age, 8.8 ± 6.32 years) were diagnosed with acute myocarditis. Fever, cough, and chest pain were the most common symptoms (68.3%, 56.7%, and 53.3%, resp.). Arrhythmia and left ventricular ejection fraction (LVEF) < 60%, vomiting, weakness, and seizure were more common in the ECMO group than in the non-ECMO group, with statistical significance (*P* < 0.05). Female sex, vomiting, weakness, seizure, arrhythmia, and echocardiography showing LVEF < 60% may predict the need for ECMO. Initial serum troponin-I cutoff values greater than 14.21 ng/mL may also indicate the need for ECMO support for children with acute myocarditis.

## 1. Introduction

Acute myocarditis, which is an inflammatory condition of the myocardium due to infection, autoimmune disease, or toxins, possibly results in life-threatening events and is relatively uncommon in children [1–3]. Clinically, viral infections are the major etiology of acute myocarditis, including enteroviruses, influenza, adenovirus, parvovirus B19, Epstein-Barr virus, human herpesvirus 6, and cytomegalovirus [4–8].

The clinical presentation of acute myocarditis may range from mild and nonspecific symptoms to fulminant cardiovascular collapse [9]. The common clinical symptoms and signs of acute myocarditis include fever, nausea and vomiting, abdominal pain, shortness of breath, tachypnea, lethargy, dysrhythmia, hypoperfusion, and shock [10–12]. The initial management of acute myocarditis includes supportive care and cardiovascular stabilization in the acute setting with the assistance of a pediatric cardiologist [13, 14]. However, if clinical deterioration progresses, left ventricular assistive devices, intra-aortic balloon pumps, and extracorporeal membrane oxygenation (ECMO) may be used as critical therapies for some patients [15, 16].

It is difficult for emergency physicians to diagnose acute myocarditis early and prognosticate correctly in children with this condition based on the clinical presentation at the time of their emergency department (ED) visit. In this study, we aimed to analyze the clinical spectrum of acute myocarditis in patients, who presented at our pediatric ED, and attempted to discover the initial clinical characteristics that could help emergency physicians to predict the outcome of acute myocarditis earlier and consult the pediatric cardiologist in a timely fashion.

## 2. Materials and Methods

### 2.1. Subjects

In this study, data were retrospectively collected on pediatric patients aged 18 years or younger who presented at our ED with a discharge diagnosis of acute myocarditis based on their medical histories. We reviewed the medical charts of all eligible patients between October 2011 and September 2016. The study was approved by the Institutional Review Board of Changhua Christian Hospital, and the necessity to obtain written consent from the participants was waived because of the retrospective nature of this study.

We identified potentially eligible patients by searching the Changhua Christian Hospital health records database. We selected the charts of all children whose International Classification of Diseases, Ninth or Tenth Revision hospitalization discharge diagnostic code was associated with myocarditis. These children had a final diagnosis of acute myocarditis at the time of discharge from the hospital or died during their hospital visit. The exclusion criteria included patients who (1) were older than 18 years, (2) did not present at the ED before admission, and (3) were not diagnosed with acute myocarditis by a pediatric cardiologist. Definite acute myocarditis is defined based on endomyocardial biopsy results according to the Dallas criteria [17]. Probable acute myocarditis was defined as acute myocarditis that was clinically determined by a pediatric cardiologist based on the patient's history, physical examination, and result of laboratory investigation (in the absence of endomyocardial biopsy). Finally, we defined all of the cases in our study as probable acute myocarditis.

The following information was obtained from the medical records of each patient: age, sex, clinical symptoms, and signs (such as fever, cough, rhinorrhea, chest pain, palpitation, diarrhea, anorexia or vomiting, tachypnea, weakness, and seizure), microbiology, electrocardiogram (ECG) and echocardiography findings, treatment modalities, complications, long-term outcomes, and laboratory tests such as white blood cell (WBC) counts, C-reactive protein (CRP), creatine phosphokinase (CK), creatine phosphokinase-MB (CK-MB), troponin-I (Trop-I), blood urea nitrogen (BUN), creatinine (Cr), alanine (ALT) and aspartate aminotransferase (AST), and serum electrolytes. We compared and analyzed the variables among the different age groups and management groups. In addition, we identified other factors that could predict acute myocarditis in children.

### 2.2. Statistical Analysis

Data of the categorical variables were analyzed with the chi-square test or Fisher's exact test, when appropriate. Continuous variables were analyzed with the Mann–Whitney *U* test and the Kruskal-Wallis test. We analyzed the factors that influenced the outcome of the patients with acute myocarditis with a univariate analysis using the Cox proportional hazards model. Distributions of variables were reported as percentages and means ± standard deviation. Receiver operating characteristic (ROC) analysis was used to evaluate the appropriate cutoff points of the biomarkers and left ventricular ejection fraction (LVEF) to assess the sensitivity (Sn) and specificity (Sp) of the predictors for ECMO management. We also examined test parameters including the area under the ROC curve (AUROC), positive likelihood ratio (LR^+^), and negative LR (LR^–^) at the various cutoff values. The AUROC, which was calculated using the trapezoidal rule, was considered a global measure of the diagnostic value of that parameter. Both LR^+^ and LR^–^ were calculated to obtain the best cutoff values. A *P* value less than 0.05 was considered statistically significant. Statistical analyses were performed with SPSS software (version 22.0, SPSS, Inc., Chicago, IL, USA).

## 3. Results

### 3.1. Demographics and Clinical Presentations

During the 5-year study period, 60 patients (32 male and 28 female; mean age, 8.8 ± 6.32 years; range, 4 months to 18 years old) who presented at the ED with probable acute myocarditis were enrolled in our series. In this study, male sex had statistical significance in the infant and adolescent groups (*P* = 0.005) (Table 1), and 33 (55%) of the 60 children were older than 6 years. However, how sex differences affect children with acute myocarditis is still unclear. The three most common symptoms and signs at presentation were fever, cough, and chest pain (68.3%, 56.7%, and 53.3%, resp.). Moreover, chest pain was a major complaint of patients in the adolescent group compared with patients in other age groups (*P* < 0.001). Palpitation was the most common symptom in the 7- to 12-year age group; however, only 16.7% of children with acute myocarditis presented with palpitation. Fever and respiratory symptoms (cough and rhinorrhea) were the most common symptoms and signs detected in the 1- to 6-year age group. Only one (1.7%) of the 60 patients had a family history of heart disease, and none of them had a history of congenital heart disease. Among these cases of acute myocarditis, only 12 patients had positive microbiological diagnoses, including three with influenza A, four with influenza B, two with enterovirus 71, one with parainfluenza type 2, one with herpes simplex virus type 1, and one with respiratory syncytial virus. Ten (16.7%) of the 60 patients were treated with ECMO and 6 (10%) died, including 2 patients not treated with ECMO. There were 6 patients placed on ECMO due to arrhythmia and 4 patients placed on ECMO due to cardiogenic shock.

### 3.2. Clinical Analysis of Patients with Acute Myocarditis Based on Whether They Underwent ECMO or Not

In our study, nine (90%) of the 10 patients diagnosed with acute myocarditis in the ECMO group were female, with statistical significance (*P* = 0.004) (Table 2). Vomiting, weakness, and seizure were more common in the ECMO group than in the non-ECMO group, with statistical significance (*P* = 0.003, *P* = 0.001, and *P* = 0.011, resp.). All of the patients in the ECMO group who underwent ECG had abnormal ECG results. In addition, arrhythmia and LVEF < 60% were more frequent in the ECMO group than in the non-ECMO group.

The differences in laboratory data between the patients with and without ECMO management are listed in Table 3. The mean serum WBC count of all patients was 11,103.17 ± 4,453.33/*μ*L (range 3,700 to 26,400/*μ*L), and 29 (48.3%) of the patients presented with leukocytosis (WBC counts > 10,000/*μ*L). The CRP levels were significantly higher in the 7- to 12-year age group (*P* = 0.003), and the mean was 71.5 ± 151.5 mg/L. In addition, the Trop-I, CK-MB, ALT, AST, BUN, Cr, calcium, and N-terminal prohormone of brain natriuretic peptide levels were significant factors that indicated the need for ECMO management. Cox proportional hazards regression analysis was then performed using the variables that had shown significant differences (*P* < 0.05) between the ECMO and the non-ECMO groups. Three parameters were associated with an increased rate of ECMO management: female sex, elevated blood CK-MB level, and LVEF < 60% (Table 4).

### 3.3. ROC Analysis of Predictors in Patients with ECMO Treatment

**Figure 1:**
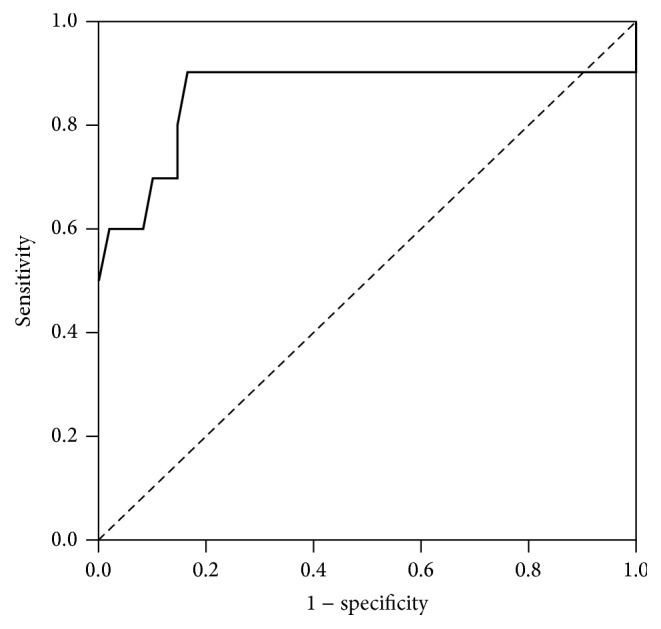
Receiver operating characteristic curve for initial left ventricular ejection fraction (LVEF) in predicting the need for extracorporeal membrane oxygenation treatment. The area under the curve was 0.86. The best cutoff value for LVEF was 57.5% (sensitivity, 0.9; specificity, 0.84).

**Figure 2:**
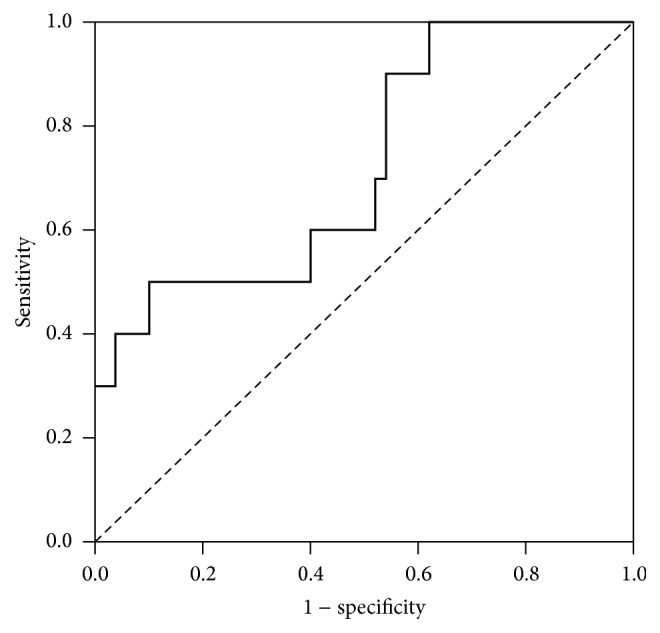
Receiver operating characteristic curve for initial serum troponin-I in predicting the need for extracorporeal membrane oxygenation treatment. The area under the curve was 0.72. The best cutoff value for serum troponin-I was 14.21 ng/mL (sensitivity, 0.5; specificity, 0.9).

The results of the ROC analysis showed that the AUROC for the initial LVEF in predicting ECMO treatment was 0.86 (Figure 1) and that the best cutoff value of the initial LVEF for predicting ECMO treatment was 57.5% (Sn, 0.9; Sp, 0.84; LR^+^, 5.51; and LR^−^, 0.12). The results of the ROC analysis showed that the AUROC for the initial serum Trop-I level in predicting the need for ECMO treatment was 0.72 (Figure 2) and that the best cutoff value of the initial Trop-I level for predicting ECMO treatment was 14.21 ng/mL (Sn, 0.5; Sp, 0.9; LR^+^, 5; and LR^−^, 5.56).

## 4. Discussion

Acute myocarditis is a relatively uncommon clinical condition in children, but the prevalence in pediatric ED patients is still unknown. During the 5-year study period, about 140,000 children presented to our pediatric ED, 60 of whom were discharged with a final diagnosis of acute myocarditis. In the preschool-age group, acute myocarditis was diagnosed more frequently in girls than in boys, but in the adolescent age group, acute myocarditis was diagnosed more frequently in boys than in girls. However, the trend of sex was not well discussed in previous studies and we cannot make a conclusion about this trend. The clinical diagnosis of acute myocarditis is challenging because the symptoms and signs are often nonspecific, especially in younger children [9]. In our series, fever (68.3%), cough (56.7%), and chest pain (53.3%) were the major symptoms in children with acute myocarditis. However, in other studies, the most common symptoms were fever, shortness of breath, gastrointestinal symptoms, hypoperfusion, and poor feeding [5, 10, 18, 19]. Furthermore, our study population had a higher incidence of cardiac symptoms (chest pain and palpitation) than other studies did [5, 10, 18, 19]. We found that chest pain was more frequent among adolescent patients; however, palpitation was more frequent in the preschool-age group. The clinical presentations of acute myocarditis seem to vary based on age, and recognizing it early remains challenging.

Delays in the diagnosis of acute myocarditis may result in increased morbidity and potential mortality. However, the diagnosis is not easy to make if the clinician is not experienced. Once acute myocarditis is suspected, further studies such as laboratory studies, chest radiography, ECG, echocardiography, cardiac magnetic resonance imaging, or endomyocardial biopsy may be clinically important to detect acute myocarditis. Acute myocarditis in children is commonly associated with severe, progressive heart failure, hospitalization, intensive care unit stays, and use of inotropic support [20]. The initial management of acute myocarditis includes supportive therapy and cardiovascular stabilization by treating heart failure and dysrhythmia, which are associated with this condition [13]. A previous study of hospitalized children in the United States showed that about half of the patients required inotropic support, 37.5% required mechanical ventilation, and 7.4% required ECMO support [21]. In our present study, 10 of 60 (16.7%) patients needed ECMO support, and 4 of them died during intensive care. The overall mortality rate in our study was 10%.

Most of the literature lists arrhythmia, end-organ failure, and circulation failure as indications for the need for ECMO support [22, 23]. Clinically, not all patients with arrhythmia or end-organ failure require prompt ECMO support; however, the circulation function of these patients may collapse at any time [22, 23]. According to a previous study, ECMO is used in about 20% of American children who are hospitalized due to myocarditis [24]. The ECMO support rate in our study was consistent with those of previous studies. Factors associated with the death of patients on ECMO support are the presence of arrhythmia during support, the need for dialysis, and higher stages of end-organ hypoperfusion, as reflected in serum lactate, creatinine, and aspartate aminotransferase levels [22, 25]. The predictors of poor outcome in children with acute myocarditis were high serum CK, appearance of ventricular tachycardia, and low LVEF in a previous study [26]. However, the factors associated with the early prediction of the need for ECMO support in children with acute myocarditis are still unclear.

In our study, we found a few factors that may predict the need for further ECMO support early in patients, including female sex, vomiting, weakness, seizure, arrhythmia, and LVEF < 60% seen on echocardiography. Moreover, elevated cardiac enzyme, abnormal liver, and renal function tests were important factors that helped us detect the need for ECMO support early in these patients. Finally, we performed a Cox proportional hazards regression analysis and found that female sex, elevated blood CK-MB level, and LVEF < 60% were important factors that predicted the need for ECMO management. Our study showed significant differences in initial LVEF detected by echocardiography between the patients with and without ECMO support, and the most appropriate cutoff value of the initial LVEF in predicting ECMO support was 57.5%. In addition, we found significant differences in serum Trop-I levels between the patients with and without ECMO support, and the most appropriate cutoff value of the initial serum Trop-I level in predicting ECMO support was 14.21 ng/mL. Therefore, primary care clinicians should be aware of these clinical factors in children with acute myocarditis to detect the need for ECMO support early. Early, aggressive treatment will be needed when children present with these risk factors.

The present study has some limitations. First, because it is a retrospective, single-center review of medical records, some details of the patients' history and physical examination may not have been rigorously documented. Second, all patients in our study were considered to have probable myocarditis due to the absence of endomyocardial biopsy results. Another limitation is that we did not study treatment details such as inotropic agent therapy or intravenous immunoglobulin therapy. Finally, the laboratory data and echocardiography may be not checked at the same time, and the definite peak and low data were difficult to record. These limitations may lead to some bias in analyzing the factors that are associated with acute myocarditis and the need for ECMO management.

## 5. Conclusions

In conclusion, pediatric acute myocarditis is relatively uncommon, but it could lead to death. Female sex, vomiting, general weakness, seizure, arrhythmia, and LVEF < 60% on echocardiography may indicate that the patient has acute myocarditis and may also increase the risk of needing ECMO support. Most importantly, a cutoff value of the initial serum Trop-I > 14.21 ng/mL and initial LVEF < 57.5% on echocardiography should be considered in children with acute myocarditis to evaluate for the need for ECMO support.

## Figures and Tables

**Table 1:** Demographics and clinical presentation of the patients with acute myocarditis.

Variables	Total (*n* = 60)		Age								*P* value
			<0		1–6		7–12		13–18		
			(*n* = 6)		(*n* = 21)		(*n* = 10)		(*n* = 23)		
	*N*	%	*N*	%	*N*	%	*N*	%	*N*	%	
Gender											
Female	28	46.7	2	33.3	15	71.4	6	60.0	5	21.7	0.005^*∗*^
Male	32	53.3	4	66.7	6	28.6	4	40.0	18	78.3	
Fever											
Yes	41	68.3	4	66.7	16	76.2	4	40.0	17	73.9	0.211
Cough											
Yes	34	56.7	3	50.0	16	76.2	3	30.0	12	52.2	0.086
Rhinorrhea											
Yes	26	43.3	3	50.0	14	66.7	2	20.0	7	30.4	0.036^*∗*^
Vomiting											
Yes	22	36.7	3	50.0	7	33.3	5	50.0	7	30.4	0.611
Diarrhea											
Yes	7	11.7	0	0.0	1	4.8	2	20.0	4	17.4	0.420
Chest pain											
Yes	32	53.3	0	0.0	6	28.6	6	60.0	20	87.0	<0.001^*∗*^
Tachypnea											
Yes	20	33.3	3	50.0	6	28.6	4	40.0	7	30.4	0.750
Palpitation											
Yes	10	16.7	0	0.0	2	9.5	6	60.0	2	8.7	0.003^*∗*^
Weakness											
Yes	19	31.7	3	50.0	6	28.6	3	30.0	7	30.4	0.788
Headache											
Yes	5	8.3	0	0.0	1	4.8	0	0.0	4	17.4	0.355
Seizure											
Yes	7	11.7	2	33.3	2	9.5	1	10.0	2	8.7	0.356
Family history of heart disease											
Yes	1	1.7	0	0.0	0	0.0	0	0.0	1	4.3	1.000

^*∗*^Statistically significant according to the *χ*^2^ test or Fisher's exact test when appropriate.

Age, years; ICU = intensive care unit.

**Table 2:** Comparison of the clinical presentations of patients with acute myocarditis based on management with ECMO or not.

Variables	No ECMO support (*n* = 50)		ECMO support (*n* = 10)		*P* value
	*N*	%	*N*	%	
Gender					
Female	19	38.0	9	90.0	0.004^*∗*^
Male	31	62.0	1	10.0	
Age					
<0	5	10.0	1	10.0	0.916
1–6	17	34.0	4	40.0	
7–12	8	16.0	2	20.0	
13–18	20	40.0	3	30.0	
Fever					
Yes	33	66.0	8	80.0	0.480
Cough					
Yes	28	56.0	6	60.0	1.000
Rhinorrhea					
Yes	23	46.0	3	30.0	0.491
Vomiting					
Yes	14	28.0	8	80.0	0.003^*∗*^
Diarrhea					
Yes	6	12.0	1	10.0	1.000
Chest pain					
Yes	29	58.0	3	30.0	0.165
Tachypnea					
Yes	16	32.0	4	40.0	0.718
Palpitation					
Yes	9	18.0	1	10.0	1.000
Weakness					
Yes	11	22.0	8	80.0	0.001^*∗*^
Headache					
Yes	4	8.0	1	10.0	1.000
Seizure					
Yes	3	6.0	4	40.0	0.011^*∗*^
ECG					
Normal	14	29.8	0	0.0	0.093
Abnormal	33	70.2	9	100.0	
Arrhythmia					
ST	18	36.0	5	50.0	<0.001^*∗*^
PSVT	6	12.0	1	10.0	
VT	1	2.0	2	20.0	
VF	0	0.0	1	10.0	
Heart block	0	0.0	1	10.0	
LVEF (%)					
<60	9	18.0	9	90.0	<0.001^*∗*^

^*∗*^Statistically significant according to the *χ*^2^ test or Fisher's exact test when appropriate.

Age, years; ECMO = extracorporeal membrane oxygenation; ECG = electrocardiogram; ST = sinus tachycardia; PSVT = paroxysmal supraventricular tachycardia; VT = ventricular tachycardia; VF = ventricular fibrillation; LVEF = left ventricular ejection fraction.

**Table 3:** Comparison of the laboratory tests of patients with acute myocarditis based on management with ECMO or not.

Variables	No ECMO support			ECMO support			*P* value
	*N*	Mean	SD	*N*	Mean	SD	
WBC (×10^9^/L)	50	11481.80	4610.77	10	9210.00	3117.85	0.148
CRP (mg/L)	35	2.62	3.60	10	6.26	11.42	0.246
Troponin-I (ng/mL)	50	4.88	10.01	10	25.48	30.95	0.026^*∗*^
CK (U/L)	34	812.87	1934.62	7	1327.14	1440.69	0.212
CK-MB (ng/mL)	38	32.68	55.16	10	122.73	163.03	0.041^*∗*^
Sodium (mmol/L)	43	137.84	3.93	10	139.20	10.65	0.599
Potassium (mmol/L)	43	3.86	0.69	10	4.32	1.78	0.480
Calcium (mg/dL)	24	8.98	0.78	9	7.17	1.42	0.001^*∗*^
AST (U/L)	18	163.78	472.55	10	291.30	208.67	0.001^*∗*^
ALT (U/L)	27	79.70	269.90	10	117.60	101.94	0.001^*∗*^
BUN (mg/dL)	20	13.90	15.99	10	25.80	14.57	0.001^*∗*^
Creatinine (mg/dL)	33	0.62	0.33	10	1.50	0.97	<0.001^*∗*^
LVEF (%)	49	66.90	9.04	10	46.10	18.61	<0.001^*∗*^
Lactate (mmol/L)	6	3.83	4.58	5	10.02	5.42	0.242
NT-proBNP (pg/mL)	8	5938.88	11977.87	5	8560.60	9730.98	0.030^*∗*^

^*∗*^Statistically significant according to the Mann–Whitney *U* test.

ECMO = extracorporeal membrane oxygenation; WBC = white blood count; CRP = C-reactive protein; CK = creatine phosphokinase; CK-MB = creatine phosphokinase-MB; AST = aspartate aminotransferase; ALT = alanine aminotransferase; BUN = blood urea nitrogen; LVEF = left ventricular ejection fraction; NT-proBNP = N-terminal prohormone of brain natriuretic peptide.

**Table 4:** Analysis of factors influencing ECMO that was performed in patients with acute myocarditis.

Variables	Total	ECMO		Univariate analysis		
		*N*	%	Hazard ratio	95% CI	*P *value
Gender						
Female	28	9	32.1	11.805	1.495–93.242	0.019^*∗*^
Male	32	1	3.1	1.000		
CK-MB						
Median (IQR)	16.5 (3.0–52.9)	56.7 (10.9–161.7)		1.006	1.002–1.011	0.004^*∗*^
LVEF (%)						
≥60	42	1	2.4	1.000		
<60	18	9	50.0	27.237	3.442–215.532	0.002^*∗*^

^*∗*^Statistically significant according to a Cox proportional-hazards regression analysis.

IQR = interquartile range; ECMO = extracorporeal membrane oxygenation; CK-MB = creatine phosphokinase-MB; LVEF = left ventricular ejection fraction.

## References

[1] Dancea A. B. (2001). Myocarditis in infants and children: a review for the paediatrician. *Paediatrics and Child Health*.

[2] Stiller B. (2008). Management of myocarditis in children: the current situation. *Advances in Experimental Medicine and Biology*.

[3] Amabile N., Fraisse A., Bouvenot J., Chetaille P., Ovaert C. (2006). Outcome of acute fulminant myocarditis in children. *Heart*.

[4] Kearney M. T., Cotton J. M., Richardson P. J., Shah A. M. (2001). Viral myocarditis and dilated cardiomyopathy: mechanisms, manifestations, and management. *Postgraduate Medical Journal*.

[5] Vashist S., Singh G. K. (2009). Acute myocarditis in children: current concepts and management. *Current Treatment Options in Cardiovascular Medicine*.

[6] Kühl U., Pauschinger M., Seeberg B. (2005). Viral persistence in the myocardium is associated with progressive cardiac dysfunction. *Circulation*.

[7] Bowles N. E., Ni J., Kearney D. L. (2003). Detection of viruses in myocardial tissues by polymerase chain reaction: evidence of adenovirus as a common cause of myocarditis in children and adults. *Journal of the American College of Cardiology*.

[8] Cooper L. T. (2009). Myocarditis. *The New England Journal of Medicine*.

[9] Simpson K. E., Canter C. E. (2011). Acute myocarditis in children. *Expert Review of Cardiovascular Therapy*.

[10] Durani Y., Egan M., Baffa J., Selbst S. M., Nager A. L. (2009). Pediatric myocarditis: presenting clinical characteristics. *American Journal of Emergency Medicine*.

[11] Ramamurthy S., Talwar K. K., Goswami K. C. (1993). Clinical profile of biopsy proven idiopathic myocarditis. *International Journal of Cardiology*.

[12] Drory Y., Turetz Y., Hiss Y. (1991). Sudden unexpected death in persons <40 years of age. *The American Journal of Cardiology*.

[13] Pettit M. A., Koyfman A., Foran M. (2014). Myocarditis. *Pediatric Emergency Care*.

[14] Maisch B., Pankuweit S. (2012). Current treatment options in (peri)myocarditis and inflammatory cardiomyopathy. *Herz*.

[15] Farrar D. J., Holman W. R., McBridge L. R. (2002). Long-term follow-up of Thoratec ventricular assist device bridge-to-recovery patients successfully removed from support after recovery of ventricular function. *The Journal of Heart and Lung Transplantation*.

[16] Moloney E. D., Egan J. J., Kelly P., Wood A. E., Cooper L. T. (2005). Transplantation for myocarditis: a controversy revisited. *Journal of Heart and Lung Transplantation*.

[17] Aretz H. T., Billingham M. E., Edwards W. D. (1987). Myocarditis: a histopathologic definition and classification. *The American Journal of Cardiovascular Pathology*.

[18] Shu-Ling C., Bautista D., Kit C. C., Su-Yin A. A. (2013). Diagnostic evaluation of pediatric myocarditis in the emergency department: a 10-year case series in the Asian population. *Pediatric Emergency Care*.

[19] Freedman S. B., Haladyn J. K., Floh A., Kirsh J. A., Taylor G., Thull-Freedman J. (2007). Pediatric myocarditis: emergency department clinical findings and diagnostic evaluation. *Pediatrics*.

[20] Canter C. E., Simpson K. P. (2014). Diagnosis and treatment of myocarditis in children in the current era. *Circulation*.

[21] Klugman D., Berger J. T., Sable C. A., He J., Khandelwal S. G., Slonim A. D. (2010). Pediatric patients hospitalized with myocarditis: a multi-institutional analysis. *Pediatric Cardiology*.

[22] Duncan B. W., Bohn D. J., Atz A. M., French J. W., Laussen P. C., Wessel D. L. (2001). Mechanical circulatory support for the treatment of children with acute fulminant myocarditis. *Journal of Thoracic and Cardiovascular Surgery*.

[23] Kato S., Morimoto S.-I., Hiramitsu S. (2004). Risk factors for patients developing a fulminant course with acute myocarditis. *Circulation Journal*.

[24] Ghelani S. J., Spaeder M. C., Pastor W., Spurney C. F., Klugman D. (2012). Demographics, trends, and outcomes in pediatric acute myocarditis in the United States, 2006 to 2011. *Circulation: Cardiovascular Quality and Outcomes*.

[25] Wilmot I., Morales D. L. S., Price J. F. (2011). Effectiveness of mechanical circulatory support in children with acute fulminant and persistent myocarditis. *Journal of Cardiac Failure*.

[26] Abe T., Tsuda E., Miyazaki A., Ishibashi-Ueda H., Yamada O. (2013). Clinical characteristics and long-term outcome of acute myocarditis in children. *Heart and Vessels*.

